# Supplementary material for: Data-Driven Mathematical Model of Apoptosis Regulation in Memory Plasma Cells
Source: Cells. 2022 May 5;11(9):1547. doi: 10.3390/cells11091547 (PMC9102437; doi:10.3390/cells11091547)
Supplement: Supplementary file 1 [file cells-11-01547-s001.zip › cells-1646604-SI.pdf]

Supplementary Figures

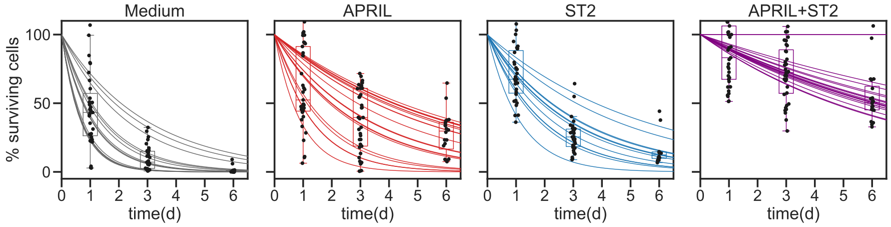

Figure S1: Plasma cell survival kinetics for individual experiments (cf. Figure 1, main text). Each dot represents one measurement for the number of surviving cells for a given time and treatment. Boxplots indicate interquartal range of observations at each time point, whiskers represent standard deviation. Each line (Grey: Medium; Red: APRIL, Blue: ST2, Purple:APRIL+ST2) represents an exponential fit for one experimental series consisting of measurements at three consecutive time points (d1, d3, d6).

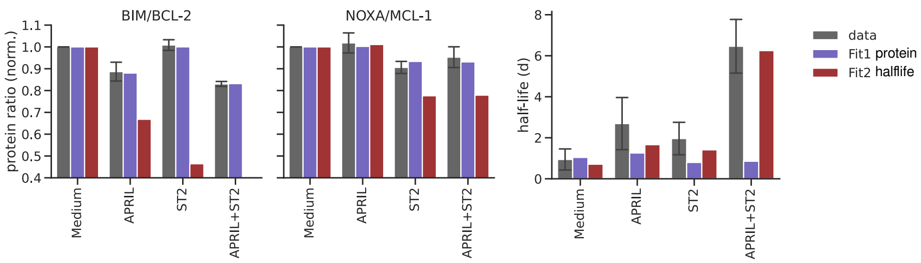

Figure S2: Protein ratio and half-life prediction give good fit results for individual fits but not for both fits combined (cf. Figure 2D-E, main text). Blue and red bars represent model fits with initial conditions that lead to good fit results for either protein ratios (blue bars) or half-lives (red bars). Available data for protein ratios and estimated half-lives shown in grey. Error bars represent standard deviation.

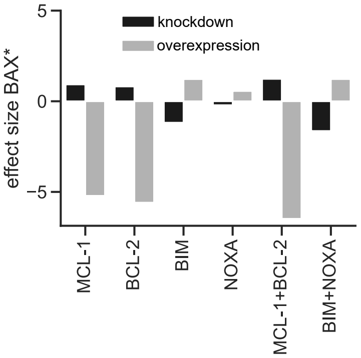

Figure S3: Effect on BAX activation for simulated knockdown or overexpression of BAX/BCL-2 family-proteins (cf. Figure 3D-E, main text). Parameters were varied by one order of magnitude with respect to the best-fit parameter value.

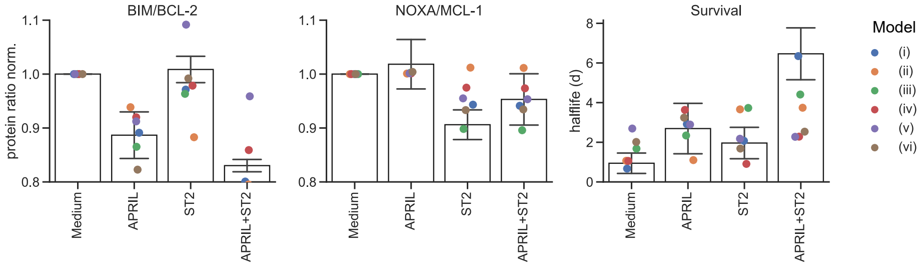

Figure S4: Model fits for protein ratio and half-life for different model versions as discussed in Figure 4, main text. Error bars represent standard-deviation.

Supplementary Text: Detailed description of the mathematical model

Modeling BAX activation

Focusing on the available data of apoptotic regulators in plasma cells, we developed an ordinary differential equations model based on mass-action-kinetics. The crucial player in our model is the pore-forming protein BAX, which upon activation facilitates the formation of the apoptotic pore. This membrane permeabilization in turn leads to the release of Cytochrome C and subsequently to the activation of downstream caspases and cell death [1–3]. For BAX activation we consider the following reactions:

$$\text{BIM} + \text{BAX} \xrightarrow{k_1} \text{BIM} + \text{BAX*} \tag{1}$$

$$\text{BAX*} + \text{BAX} \xrightarrow{k_1} 2\text{BAX*} \tag{2}$$

$$\text{BAX*} \xrightarrow{k_2} \text{BAX} \tag{3}$$

With k_1 and k_2 denoting BAX activation and deactivation respectively. Additionally, we considered here direct activation of BAX via the proapoptotic protein BIM [4]. For further regulation of BAX activation, we considered antiapoptotic proteins BCL-2 and MCL-1 that have been shown to directly bind and form complexes with BAX, thereby inhibiting apoptosis [5]. Moreover, we considered pro-apoptotic proteins NOXA and BIM that have been shown to form heterodimers with anti-apoptotic proteins. Hence, the proapoptotic proteins prevent BAX deactivation by inhibiting the antipoptotic proteins [5]. For complex formation, we therefore consider:

$$\text{MCL-1} + \text{BIM} \underset{k_3^-}{\overset{k^+}{\rightleftharpoons}} \text{MCL-1:BIM} \tag{4}$$

$$\text{MCL-1} + \text{NOXA} \underset{k_4^-}{\overset{k^+}{\rightleftharpoons}} \text{MCL-1:NOXA} \tag{5}$$

$$\text{MCL-1} + \text{BAX*} \underset{k_5^-}{\overset{k^+}{\rightleftharpoons}} \text{MCL-1:BAX*} \tag{6}$$

$$\text{BCL-2} + \text{BIM} \underset{k_6^-}{\overset{k^+}{\rightleftharpoons}} \text{BCL-2:BIM} \tag{7}$$

$$\text{BCL-2} + \text{BAX*} \underset{k_7^-}{\overset{k^+}{\rightleftharpoons}} \text{BCL-2:BAX*} \tag{8}$$

Here k^+ and k_i^- denote the complex association and dissociation rate respectively.

Modeling input of APRIL and ST2 on BAX activation

We assumed that input signals APRIL and ST2 affect the steady-state concentration of pro- and anti-apoptotic proteins which in turn modulate the availability of activated BAX. Specifically, we assumed that each protein in the network is subject to growth and decay in the simple form

$$\frac{dx_P}{dt} = g_P(\text{APRIL,ST2}) - d_P x_P, \tag{9}$$

where APRIL and ST2 affect the growth rate g_P. We modeled the growth rate dependency of the inputs as:

$$g_{\text{P}} = g_{\text{P},0}\Big(1 + a_P f([\text{APRIL}]) - s_P f([\text{ST2}])\Big), \tag{10}$$

with

$$f(x) = \begin{cases} 1 & \text{if } x > 0 \\ 0 & \text{otherwise.} \end{cases} \tag{11}$$

Here, a_P and s_P are the maximal effects of APRIL and ST2 on the growth rate. Since it has been previously shown that ST2 inhibits FoxO, we assume an inhibitory effect for ST2. As described in the main text, we designed the network based on our observations that APRIL only affected BCL-2 whereas ST2 affected BIM, NOXA and MCL-1. Further, we modeled reversible formation of heterodimeric complexes according to:

$$\frac{d[XY]}{dt} = k^+[X][Y] - k^-[XY] - (d_X + d_Y)[XY]. \tag{12}$$

Here, k^+ is the reaction rate with which the complex is formed and k^- is the reaction rate with which the complex dissociates. For the protein species in our model, quantitative estimates can be mainly found for the dissociation constant K_D. Therefore, we chose to set the complex formation rate k^+ to a fixed value (see Table 2, main text) and calculated the complex dissocation rate as

$$k_i^- = K_{D,i} \cdot k^+. \tag{13}$$

Taken together, this results in the following set of ordinary differential equations

(Abbreviations: A: MCL-1; B: BCL-2; C: BIM; D: NOXA; X: BAX; Z: BAX*):

$$\frac{dA}{dt} = g_A - d_A[A] - k^+[A][C] - k^+[A][Z] - k^+[A][D] + k_3^-[AC] + k_5^-[AZ] + k_4^-[AD] \tag{14}$$

$$\frac{dB}{dt} = g_B - d_B[B] - k^+[B][C] - k^+[B][Z] + k_6^-[BC] + k_7^-[BZ] \tag{15}$$

$$\frac{dC}{dt} = g_C - d_C[C] - k^+[A][C] - k^+[B][C] + k_3^-[AC] + k_6^-[BC] \tag{16}$$

$$\frac{dD}{dt} = g_D - d_D[D] - k^+[A][D] + k_4^-[AD] \tag{17}$$

$$\frac{dX}{dt} = g_X - d_X[X] - k_1([C] + [Z])[X] + k_2[Z] \tag{18}$$

$$\frac{dZ}{dt} = -d_X[Z] + k_1([C] + [Z])[X] - k_2[Z] - k^+[A][Z] - k^+[B][Z] + k_5^-[AZ] + k_7^-[BZ] \tag{19}$$

$$\frac{d[AC]}{dt} = -(d_A + d_C)[AC] + k^+[A][C] - k_3^-[AC] \tag{20}$$

$$\frac{d[AD]}{dt} = -(d_A + d_D)[AD] + k^+[A][D] - k_4^-[AD] \tag{21}$$

$$\frac{d[AZ]}{dt} = -(d_A + d_Z)[AZ] + k^+[A][Z] - k_5^-[AZ] \tag{22}$$

$$\frac{d[BC]}{dt} = -(d_B + d_C)[BC] + k^+[B][C] - k_6^-[BC] \tag{23}$$

$$\frac{d[BZ]}{dt} = -(d_B + d_Z)[BZ] + k^+[B][Z] - k_7^-[BZ] \tag{24}$$

References

1. Westphal, D., Dewson, G., Czabotar, P. E. & Kluck, R. M. Molecular biology of BAX and BAK activation and action. *BBA Mol. Cell Res.* **1813,** 521–531 (2011).
2. Westphal, D., Kluck, R. M. & Dewson, G. Building blocks of the apoptotic pore: How BAX and BAK are activated and oligomerize during apoptosis. *Cell Death Differ.* **21,** 196–205 (2014).
3. Chen, C., Cui, J., Lu, H., Wang, R., Zhang, S. & Shen, P. Modeling of the role of a BAX-activation switch in the mitochondrial apoptosis decision. *Biophys. J.* **92,** 4304–4315 (2007).
4. Du, H., Wolf, J., Schafer, B., Moldoveanu, T., Chipuk, J. E. & Kuwana, T. BH3 domains other than BIM and BID can directly activate BAX/BAK. *J. Biol. Chem.* **286,** 491–501 (2011).
5. Czabotar, P. E., Lessene, G., Strasser, A. & Adams, J. M. Control of apoptosis by the BCL-2 protein family: Implications for physiology and therapy. *Nat. Rev. Mol. Cell Biol.* **15,** 49–63 (2014).
